# Characterization of affective states by pupillary dynamics and autonomic correlates

**DOI:** 10.3389/fneng.2013.00009

**Published:** 2013-11-06

**Authors:** Francesco Onorati, Riccardo Barbieri, Maurizio Mauri, Vincenzo Russo, Luca Mainardi

**Affiliations:** ^1^Dipartimento di Elettronica, Informazione e Bioingegneria, Politecnico di MilanoMilan, Italy; ^2^Massachusetts Institute of TechnologyCambridge, MA, USA; ^3^Massachusetts General HospitalBoston, MA, USA; ^4^Behavior and Brain Lab, IULM UniversityMilan, Italy

**Keywords:** pupil dilation (PD), heart rate variability (HRV), autonomic nervous system (ANS), emotions, reconstruction, spectral analysis, very high frequencies, coherence analysis

## Abstract

With the recent advent of new recording devices and an easier access to signal processing tools, researchers are increasingly exploring and studying the Pupil Dilation (PD) signal. Recently, numerous studies pointed out the relations between PD dynamics and psychophysiological states. Although it is well known that PD is controlled by the Autonomic Nervous System (ANS), and ANS responses are related to emotional events/stimuli, the relationship between emotional states and PD is still an open issue. The aim of this study is to define the statistical properties of the PD signal, to understand its relation with ANS correlates such as Heart Rate Variability (HRV) and respiration (RESP), and to explore if PD could provide information for the evaluation of the psychophysiological response of ANS to affective triggering events. ECG, RESP, and PD data from 13 normal subjects were recorded during a memory recall paradigm, and processed with spectral and cross-spectral analysis. Our results demonstrate that variability indices extracted from fast PD oscillations, not observable through standard cardiorespiratory identification in the frequency domain, would be able to discern psychophysiological responses elicited by basic emotional stimuli. A strong linear coupling was found between the variables, due to the influence of RESP on both PD and HRV within the High Frequency (HF) band, from 0.15 to 0.45 Hz. Most importantly, our results point at PD features as possible candidates for characterizing basic emotional stimuli.

## 1. Introduction

The Autonomic Nervous System (ANS) primarily innervates the smooth musculature of all organs, the heart and the glands, and mediates the neuronal regulation of the internal environment to keep a proper balance, a process in general not under direct voluntary control (Jänig, 1989). The existence of a relation between psychophysiological states and ANS activity has been widely documented (Ekman et al., 1983; Levenson et al., 1990; Collet et al., 1997; Christie and Friedman, 2004). On the other hand, Cacioppo et al., (2000) performed a meta-analysis of physiological responses to affective states and claimed that the scientific literature has presented inconclusive evidence-based results supporting the existence of specific patterns of peripheral activity as effected by emotional stimuli. However, it has been shown that ANS indices show specific activation during particular emotional events, and negative emotions are in general associated with more evident bodily responses when compared with positive ones. More recently, Rainville et al., (2006) showed that, under specific experimental conditions, it would be possible to differentiate emotions. Moreover, Stephens et al., (2010) supported the hypothesis that patterns of autonomic correlates, rather than single measurements, would lead to more specific and discernible emotional responses. Although these findings address a link between ANS and emotional events, it is still a debated question whether the ANS activity is driven by cognitive elaboration of the emotional event (James, 1894; Damasio, 1994; Levenson, 2003) or vice versa (Cannon, 1927; Arnold, 1960; Schachter and Singer, 1962). For a complete review of the recent contributions to the debate, see Lowe and Ziemke (2011).

The Sympathetic Nervous System (SNS) and the Parasympathetic Nervous System (PNS) both innervate the heart (Berntson et al., 1997). The Electrocardiogram (ECG) is one of the most distinct and accessible signal related to the heart function, widely used for psychophysiological purposes (Cacioppo et al., 2007). From the ECG is possible to define the RR series, which is the series of the time intervals of consecutive R-waves (Camm et al., 1996). The elicited heartbeat variations of the RR series have been defined as Heart Rate Variability (HRV) and extensively studied in the last decades (Camm et al., 1996). An important element driving HRV is Respiratory Sinus Arrhythmia (RSA). RSA is a natural variation in heart rate due to respiratory influences, mediated by vagal cardiac nerve (Katona and Jih, 1975). RSA provides an indirect and non-invasive measure of parasympathetic cardiac control, typically occurring in the High Frequency (HF) band in the frequency-domain transform of the RR series. Slow HRV oscillations in the Low Frequency (LF) band are influenced by both vagal and sympathetic activity (Berntson et al., 1997).

One of the most recent ANS correlate introduced in scientific literature is Pupil Dilation (PD) (Beatty and Lucero-Wagoner, 2000; Lanata et al., 2011). The pupillary response, whose neural pathways are mediated by the ANS, is determined by the activity of two smooth iris muscles: PNS innervates the *sphincter pupillae* and controls the pupillary constriction, whereas SNS causes the excitation of the *dilator pupillae* (Beatty and Lucero-Wagoner, 2000). The major role of these muscles is to adjust the amount of light allowed to enter the eye according to the surrounding illumination level, a phenomenon called Pupillary Light Reflex (Ellis, 1981), the most widely studied and reported PD function. On the other hand, spontaneous pupillary fluctuations (SPF) occur in permanent lighting and eye fixation conditions as well. SPF represent a dynamical equilibrium modulated by autonomous and central nervous systems (Nowak et al., 2008), and have been shown to reflect cognitive and affective processes (Andreassi, 2000). Borgdorff (1975), after having performed experiments in which he showed the existence of respiratory pupil fluctuations in cats, proposed a physiological model by which the respiratory and pressure signals influence PD dynamics. Different pupillary responses were observed with respect to different cognitive stimuli (Steinhauer et al., 2004). Calcagnini et al., (2000), after having performed a cross-spectral analysis, reported that baroreceptor-sensitive fluctuations are visible in PD dynamics during a Head-Up Tilt test. The relation between PD dynamics and psychophysiological states was pointed out first by Partala and Surakka, (2003) and subsequently by Bradley et al., (2008). Both these studies reported that variations of PD are related to autonomic activation during affective processing. However, PD fluctuations during emotional events have not been adequately explored.

The aim of this study is to apply mathematical methods to process PD dynamics during an emotionally characterized protocol in order to define descriptive statistical indices of PD and to explore their relation with ANS correlates such as HRV and respiration (RESP). In addition, we verify if PD could provide information in the evaluation of ANS responses during a psychophysiological affective protocol.

## 2. Materials and methods

### 2.1. Experimental protocol

Personal feelings are proved to produce large responses in measurements based on self-reports or physiological features (Bond, 1998; Mauri et al., 2010). The presented experimental protocol is inspired by previous works (Rainville et al., 2006; Mauri et al., 2012), in which a memory recall paradigm of emotionally characterized autobiographical episodes was successfully used to trigger the physiological response of the ANS.

The target emotions were chosen within the classical discrete categorical model of emotions, according to which they are represented as discernible but fuzzy bounded entities (Russell, 1980; Lang, 1995; Valenza et al., 2012). We selected promptly understandable emotions to avoid to mislead the subjects. Moreover, the arousal and valence^1^ values of the target emotions should have been as high as possible. Therefore, the target emotions considered in this work are “Happiness,” “Sadness,” and “Anger.” They are compared with a reference event, i.e., a resting period termed “Baseline.”

Healthy volunteers were recruited from the student body of IULM University of Milan. The subjects did not suffer from mental pathologies. The experimental protocol was divided in two phases. In the first phase, subjects were scheduled for an interview where they were asked to recall and loudly tell two recent autobiographical episodes for each of the target emotions. Then the psychologist, in agreement with the subject, chose the most vivid and intense episode for each target emotion. These episodes are then used in the second phase, as described below. In Table 1 we report the most common episodes recalled by the subjects, for each target emotion.

**Table 1:** **Descriptions of the most common autobiographical episodes the subjects recalled during the first phase of the protocol for each target emotion**.

**Target emotion**	**Episodes**
Happiness	To meet a close relative, a friend, or partner after a long time; an important sport success
Anger	To be cheated on; to fail a test or a school exam
Sadness	The grief for the death of a close relative or a friend; the end of a love affair

Subjects who could not recall vivid recent episodes for each of the target emotions were excluded from the second phase of the experimental protocol. In total, 13 subjects participated in the second phase of the experiment: they were scheduled for a second appointment and admonished not to consume coffee or caffeinated products at least 4 h before it.

The second phase of the experiment is the recording session, during which the subjects were helped in recalling the same autobiographical episodes chosen with the psychologist during the first phase of the experiment.

**Figure 1:**
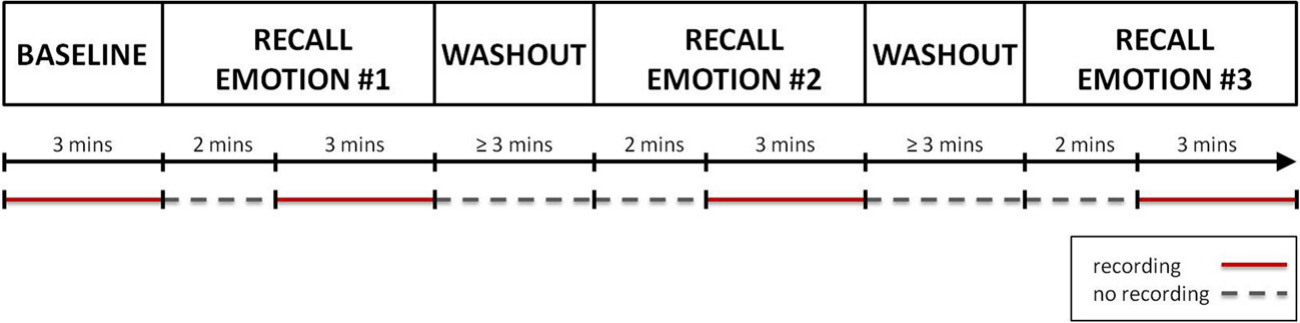
**A graphical representation of the protocol.** After the recording of the “Baseline,” the recall of the first autobiographical episode could start. For about 2 min the subject was helped in recalling the emotional even. Once he/she re-experienced the target emotion, a 3 min long recording of physiological data was performed. Finally a washout period of at least 3 min was provided before starting the recall of the next emotional episode. The same procedure was repeated for each target emotion. The recording phases are indicated by red segments, while the phases during which no recording was performed are indicated by gray dotted segments.

Figure 1 shows a graphical representation of the recording session protocol. The session started with a 3 min long “Baseline” condition, during which the subject was instructed to sit quietly and to clear his/her mind of thoughts, feelings, and memories. Hereafter, the recall of the autobiographical episodes could begin. For each target emotion, the psychologist drove the subject in the recalling of the autobiographical episode. This phase lasted approximately 2 min. When the subject confirmed he/she was re-experiencing the emotion, the psychologist asked him/her to continue the recall of the episode, to refrain from speaking and to keep the gaze on the monitor for the next 3 min, during which the physiological data were recorded. After the recall, a washout period of at least 3 min was provided before starting the recall of the next emotional episode. During the washout period the subject was asked to relax and to clear again his/her mind. The sequence of the emotional episodes to recall was randomly sorted for each subject.

### 2.2. Physiological measures

The subjects were asked to sit in front of the SensoMotoric Instruments RED250™ Eye-tracker monitor provided by a color gray screen at a fixed distance of 70 cm, in a room with constant illumination conditions. The PD signals were recorded at a sample frequency of 250 Hz; prior to start the “Baseline” and the single emotion recalls, calibration of the eye-tracker was performed. For computation purposes, the signals were then low-passed and resampled at 50 Hz.

ECG and the RESP signals were recorded using a Flexcomp Infinity™ encoder (Thought Technology Ltd.; Montreal, Canada) at a sampling rate of 2048 Hz, then resampled at 256 Hz. Relative changes in thoracic expansion were measured using a band provided with a tension-sensitive latex transducer; the thoracic band was placed over the upper part of the chest, individually adjusted to produce the maximal deflection during normal breathing; in the pre-experimental phase the subject was asked to exhale and inhale in a sealed reservoir bag: this procedure was designed to calibrate the RESP signal and to cancel the effects of the differences due to the band positioning and to the different thoracic expansions among the subjects. ECG was recorded using a standard 3 leads montage (Einthoven lead 2 configuration) on the right and left forearms. R-waves were detected and corrected from ectopic beats with a specific detection and correction program (Citi et al., 2012). Physiological data were not continuously recorded during the second phase of the experiment. The recording epochs are highlighted in Figure 1.

### 2.3. Pupil dilation analysis

Before performing the PD Analysis, a PD Reconstruction phase was needed to fill the missing data due to eye-blinking events and artifacts, to obtain an evenly sampled signal. Eye-blinking events were automatically recognized by the eye-tracker and reviewed offline to correct misdetection or missed events. A temporal window from 100 ms before to 100 ms after each eye-blinking event was clipped from the data (Einhäuser et al., 2008), and a gap of missing data in the PD series was created. These missing segments were then reconstructed before further computation. The reconstructed signal was then analyzed in both time and frequency domain, to extract the characteristic features.

#### 2.3.1. Pupil dilation reconstruction

An iterative method based on Singular Spectrum Analysis (SSA), called Iterative-SSA, was implemented to fill the gap generated on a PD signal by blink events (Sassi et al., 2009; Onorati et al., 2012).

The SSA is a powerful signal processing technique introduced by Broomhead and King (1986) to decompose the original series into a sum of independent and interpretable components such as slowly varying trend, oscillatory components and structureless noise. In summary, the algorithm embeds a one-dimensional time series ***x***(*t*_*n*_) in a *M*-dimensional vector series.

Once the dimension *M* for the embedding is chosen, the *M*-lag correlation matrix **C_*x*_** is computed from the time series ***x***(*t*_*n*_) as

(1) $$\begin{array}{c} C_{x}=\frac{1}{N-\left|i-j\right|}\sum_{n=1}^{N-\left|i-j\right|}x(t_{n})x(t_{n+\left|i-j\right|})\text{with }0\leq \left|i-j\right|<M. \end{array}$$

A singular value decomposition (SVD) is carried on **C_*x*_** and hence the eigenvectors, or Empirical Orthogonal Functions (EOF), are obtained. The time-series ***x***(*t*_*n*_) is projected onto them, producing *M* principal components **PC** of length (*N* − *M* + 1).

(2) $$\begin{array}{c} PC(t_{n})=\sum_{j=1}^{M}x(t_{n}+j)EOF_{l}(j)\text{ with }0\leq n\leq N-M, \end{array}$$

The original time series is expanded in an optimal way as the sum of its *M* reconstructed components **RC**(*t*_*n*_) (Vautard et al., 1992), defined as

(3) $$RC(t_{n})=\frac{1}{M}\sum_{k=1}^{M}PC(t_{n}-k)EOF(k).$$

The choice of *M* is a key problem. Since dynamics with periods longer than *M* cannot be solved, the greater *M*, the longer are the time intervals that can be reconstructed. Moreover, the spectral resolution is limited to 1/*M*, which suggests the choice of a *M* as large as possible. On the other hand, a *M* too large would cause the splitting of a single component in two or more components (Vautard et al., 1992) and increase computational burden.

For the estimation of missing data of the time series, Schoellhamer (2001) and Sassi et al. (2009) suggested to set *M* to the width of the gaps to be filled. Therefore *M* was set to the maximum width of the gap in the series (including not only eye-blinking events but also artifacts due to misalignment between pupil and sensor) which resulted of width *L*_gap_ = 3 s (on average across subjects), that is *M* = *f*_*s*_ · *L*_gap_ = 150, where *f*_*s*_ is the sampling frequency.

**Figure 2:**
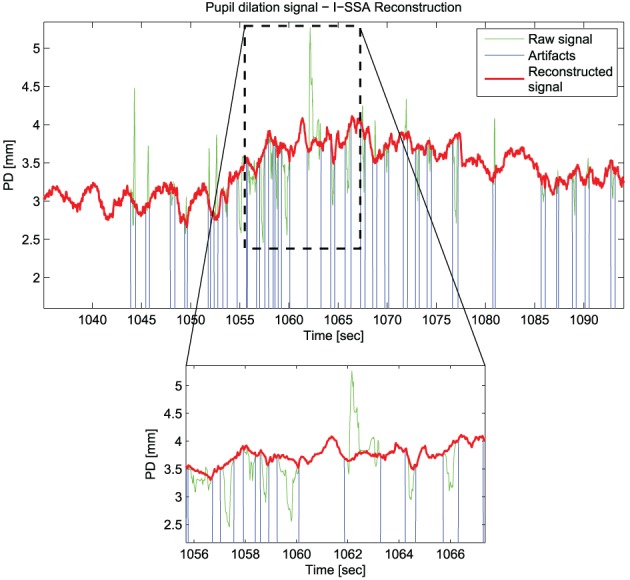
**An example of the reconstruction of Pupil Dilation signal with the Iterative-SSA algorithm for the subject “sbj15,” during “Sadness”**.

Figure 2 shows an example of the reconstruction performed using the Iterative-SSA algorithm.

#### 2.3.2. Time domain analysis

As previous studies reported a spectral content for the PD signal up to 4–5 Hz (Nakayama and Shimizu, 2004), we low-passed and resampled the reconstructed PD signal at 10 Hz. Temporal aggregate statistical indices were computed: the mean value μ of the PD signal, its standard deviation σ and the coefficient of variation *c*_*v*_, i.e., the ratio between the standard deviation σ and mean μ.

#### 2.3.3. Spectral analysis

A parametric spectral analysis, via autoregressive (AR) model coefficients estimation was performed to compute the spectral components. The order of the model was chosen according to the Akaike Information Criterion (AIC) (Akaike, 1974) and a spectral decomposition procedure was applied to calculate each component of the the Power Spectral Density (PSD) of the signal (Zetterberg, 1969). We referred to the standard measurements of HRV generally used in both psychophysiological and clinical settings (Camm et al., 1996). The power of each PD rhythm was summed within the corresponding frequency bands, i.e., LF, from 0.04 to 0.15 Hz, and HF, from 0.15 to 0.45 Hz (Camm et al., 1996). To explore the frequency contributions from 0.45 up to 5 Hz, which we term as very high frequency (VHF), a high-pass filter was applied with a cutoff frequency at f_c_ = 0.2 Hz, to eliminate the dominant contributes from lower frequency content. Based on the observation of the PSD high frequency contents, we considered the following frequency bands: [0.45–1], [1–2.5], and [2.5–5] Hz, termed, respectively as VHF_[0.45–1]_, VHF_[1–2.5]_, and VHF_[2.5–5]_. Absolute powers were computed for each band.

#### 2.3.4. Cross-spectral analysis

Given two time series *x*_1_(*t*) and *x*_2_(*t*) of length *N*, a system describing their mutual interactions can be expressed in the form of a bivariate autoregressive (AR) model

(4) $$X(t)=-\sum_{k=1}^{p}A(k)X(t-k)+w(t),$$

where **X**(*t*) is the vector time series [***x***_1_(*t*) ***x***_2_(*t*)], *p* is the order of the model, ***w***(*t*) is a vector of white noises and **A** is the matrix of the AR coefficients, estimated along with the covariance matrix Σ of the input noise

(5) $$\Sigma =\left[\begin{array}{c} \sigma_{11}^{2}\sigma_{12}\\ \sigma_{21}\sigma_{22}^{2} \end{array}\right].$$

To compute ***w***(*t*), **A** and Σ we used the Levinson–Wiggins–Robinson algorithm (Wiggins and Robinson, 1965; Barbieri et al., 2001) to solve the Yule–Walker equations. The order of the bivariate model was chosen according to the Akaike Information Criterion (AIC) (Akaike, 1974).

Once the AR coefficients and the covariances are obtained, it is possible to estimate the cross-spectral matrix **S**(*f*) as

(6) $$\begin{array}{c} S(f)=H\Sigma H^{\text{H}}\;H(f)={\left(I-A(f)\right)}^{-1} \end{array}.$$

where the superscript H indicates Hermitian transpose and **A**(*f*) is the Fourier Transform of **A**(*k*).

Estimated the auto-spectra *S*_***x*_1_**_(*f*) and *S*_***x*_2_**_(*f*) and the cross-spectrum *S*_***x***_1_, ***x***_2__(*f*), it is possible to compute the Coherence γ^2^(*f*) as

(7) $$\gamma^{2}(f)=\frac{{\left|S_{x_{1},x_{2}}(f)\right|}^{2}}{S_{x_{1}}(f)S_{x_{2}}(f)},$$

and the normalized squared Directed (Causal) Coherence (nDC_*ji*_) from channel *i* to channel *j* as

(8) $$\text{nDC}{\left(f\right)}_{ji}=\frac{{\left|H_{ji}(f)\right|}^{2}}{S_{jj}(f)}.$$

Finally, according to a bivariate closed-loop model (Barbieri et al., 2001), the causal gains *G*_*i → j*_ are calculated as

(9) $$\text{G}{\left(f\right)}_{i\to j}=\left|\frac{A{\left(f\right)}_{ji}}{1-A{\left(f\right)}_{jj}}\right|.$$

PD signals were sampled at the occurrences of the R-waves. The Coherence functions γ^2^ between the signals were computed by Equations (6) and (7).

For assessing the significance zero level of the Coherence (and for nDC) a surrogate data analysis procedure was performed (Faes et al., 2004, 2009): *N* = 200 couples of surrogate series (Schreiber and Schmitz, 2000) were generated from the original series via an Unwindowed Fourier Transform (UFT) algorithm (Theiler et al., 1992); the aim of the UFT surrogate procedure is to preserve the spectrum information of each time series, but to completely destroy the phase correlation between them; the Coherence and the nDC were then computed between each of the 200 pairs of surrogate series and the relative sampling distributions were obtained at each frequency; the zero thresholds γ^2^_θ_ and nDC_*ji*, θ_ were set at the 95th percentile at each frequency.

The corresponding value of causal gains are evaluated at the frequency values of the peaks of nDC.

#### 2.3.5. Statistical analysis

We computed all the above indices from segments long at least 90 s for the available signals (PD, RR, RESP) during the “Baseline” condition and the three emotional events “Happiness,” “Anger” and “Sadness”. For each index and epoch, we performed a Lilliefors test (Lilliefors, 1967), to verify if the hypothesis of normality could not be rejected. We chose to perform non-parametric tests because of the low number of the subjects, and the presence of high variance and possible outliers on data, as obtained performing a Grubbs' test (Grubbs, 1969). For the analysis of variance we performed a Friedman test (Friedman, 1937). As *post-hoc* analysis, we performed the Wilcoxon signed rank test (Wilcoxon, 1945), to test the differences between the “Baseline” and each emotional event. For the analysis of Gains, we used the Kruskall–Wallis one-way test. To offset the impact of multiple comparisons, a Bonferroni correction was applied to the level of significance: as we were interested only in differences between “Baseline” and the other psychophysiological conditions, and considering the level of significance for the whole family of tests α_family_ = 0.05, the level of significance of each individual test is α = α_family_/n, where n = 3.

#### 2.3.6. Discriminant analysis

We performed a discriminant analysis to test the ability of the most relevant indices to potentially distinguish “Baseline”from the emotionally characterized events. We computed true positive rate (TPR) and false positive rate (FPR) varying the discriminant threshold. Statistical indices such as Sensitivity (*Se* = TPR) and Specificity (*Sp* = 1-FPR) were then obtained.

## 3. Results

### 3.1. Preliminary statistics of the pupil dilation signal

Table 2 shows the temporal PD indices obtained on the analyzed population.

**Table 2:** **Mean values and standard deviations (mean ± standard deviation) of temporal indices of PD**.

	**Baseline**	**Happiness**	**Anger**	**Sadness**
μ	3.998 ± 0.397	3.981 ± 0.451	3.916 ± 0.498	3.990 ± 0.460
σ	0.282 ± 0.057	0.240 ± 0.051	0.237 ± 0.073	0.239 ± 0.070
*c*_*v*_	0.071 ± 0.015	0.061 ± 0.013	0.060 ± 0.017	0.060 ± 0.015

We observe a decrease of the overall variability during triggering events. The reduction of σ, although not significant, occurs during emotional events regardless of mean pupil size μ, which is possibly dependent on other causes, such as accommodation or brightness level. As a consequence, the *c*_v_ is higher for every emotionally characterized events. However, Friedman test did not show any inter-groups effect.

### 3.2. PD and cardiorespiratory ANS correlates

#### 3.2.1. Spectral analysis

Table 3 reports the obtained data for cardiorespiratory indexes.

**Table 3:** **Mean values and standard deviations (mean ± standard deviation) of cardiorespiratory indices**.

	**Baseline**	**Happiness**	**Anger**	**Sadness**
**RR**				
μ_RR_	0.826 ± 0.115	0.811 ± 0.111	0.805 ± 0.121	0.797 ± 0.104
LF norm	0.453 ± 0.139	0.532 ± 0.250	0.588 ± 0.246	0.570 ± 0.185
HF norm	0.418 ± 0.203	0.352 ± 0.280	0.271 ± 0.248	0.260 ± 0.243
LF/HF	1.281 ± 0.556	4.550 ± 9.251	8.370 ± 14.070	5.775 ± 6.998
**RESP**				
*f*_RESP_	0.271 ± 0.060	0.262 ± 0.093	0.267 ± 0.092	0.241 ± 0.110
σ^2^	0.012 ± 0.023	0.020 ± 0.038	0.020 ± 0.042	0.028 ± 0.059
HF_%_	0.765 ± 0.157	0.656 ± 0.281	0.752 ± 0.188	0.536 ± 0.278

The Friedman test didn't show any statistical significance among the psychophysiological conditions in the cardiorespiratory features presented in Table 3. The trends of the considered indices reveal a highest sympathetic activation (vagal withdrawal) for the emotional events: an increase in the LF component was observed simultaneously to a decrease in the HF component. Accordingly, an increase in the LF/HF ratio was observed.

PD spectral indices at both low frequencies (LF and HF) and high frequencies (VHF) are shown in Table 4 for all the experimental conditions.

**Table 4:** **Mean values and standard errors for all the different spectral indices of the PD**.

**PD**	**Baseline**	**Happiness**	**Anger**	**Sadness**
LF_PD_	0.030 ± 0.023	0.023 ± 0.017	0.026 ± 0.026	0.019 ± 0.013
LF_PD_ norm	0.467 ± 0.189	0.465 ± 0.148	0.490 ± 0.213	0.459 ± 0.161
HF_PD_	0.016 ± 0.010	0.012 ± 0.007	0.010 ± 0.007^*^	**0.010 ± 0.007**^†^
HF_PD_ norm	0.313 ± 0.223	0.319 ± 0.214	0.216 ± 0.075	0.275 ± 0.160
LF/HF_PD_	2.268 ± 1.677	2.851 ± 3.336	3.019 ± 3.486	2.400 ± 2.141
VHF_[0.45–1]_(^*^10^−2^)	0.464 ± 0.192	0.486 ± 0.378	0.374 ± 0.260	0.425 ± 0.293
VHF_[1–2.5]_(^*^10^−2^)	0.204 ± 0.115	0.204 ± 0.204	0.158 ± 0.137	0.208 ± 0.182
VHF_[2.5–5]_(^*^10^−3^)	0.484 ± 0.174	0.442 ± 0.190	**0.348 ± 0.212**^†^	0.409 ± 0.214

A asterisk (

* )indicates a p-value < 0.05, while a dagger (

† ) indicates a p-value < 0.016. Statistically significant differences were bold typed.

**Figure 3:**
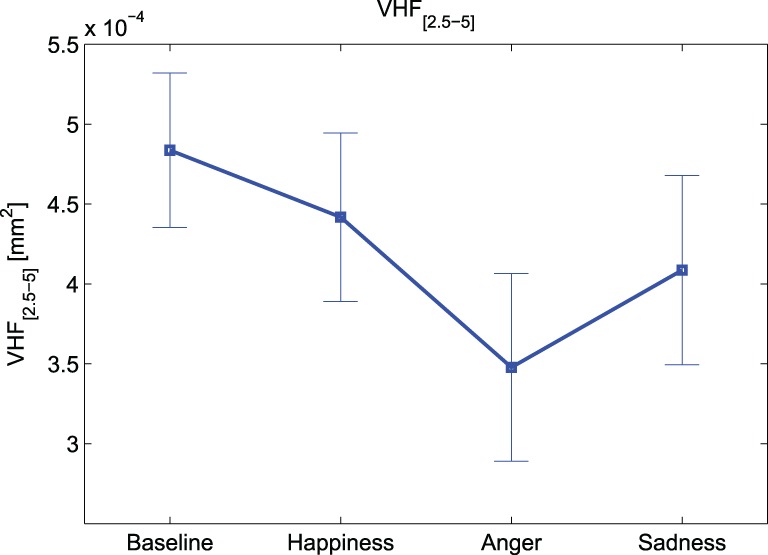
**Means and standard errors of VHF_[2.5–5]_**.

Notably, according to the Friedman test, there are significant inter-group differences due to the different experimental conditions for HF_PD_ [*F*_(3, 36)_ = 3.943, *p*-value < 0.05], VHF_[0.45–1]_ [*F*_(3, 36)_ = 3.943, *p*-value < 0.05] and VHF_[2.5–5]_ [*F*_(3, 36)_ = 3.457, *p*-value < 0.05] indices. Overall we can observe a decrease in total power, as well as in each frequency band, for all emotional events. As it is depicted in Figure 3, at VHF_[2.5–5]_ this trend is more marked, showing a highly significant difference (*p*-value < 0.016) between “Baseline” and “Anger”.

**Figure 4:**
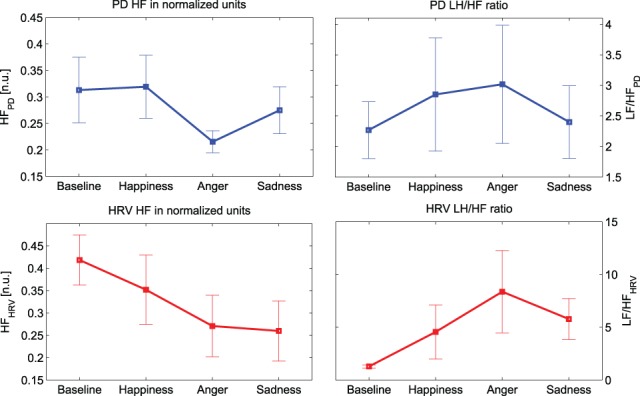
**Mean values and standard errors of the HF power (left) LF/HF ratio (right) for both PD (plots at the top, in blue) and HRV (plots at the bottom, in red)**.

Similarly, the absolute power in both LF and HF bands shows a clear decrease. In particular, this decrease is statistically significant (*p*-value < 0.016) for “Anger” at HF. It can be noticed that the trends of the PD power indices in normalized units show similar behavior to their cardiac counterpart, as shown in Figure 4. There is an evident decrease in the HF power in normalized units and an increase of the LF/HF ratio during the emotional events, particularly during “Anger,” although there is no significant difference among these PD indices.

#### 3.2.2. Coherence analysis

**Figure 5:**
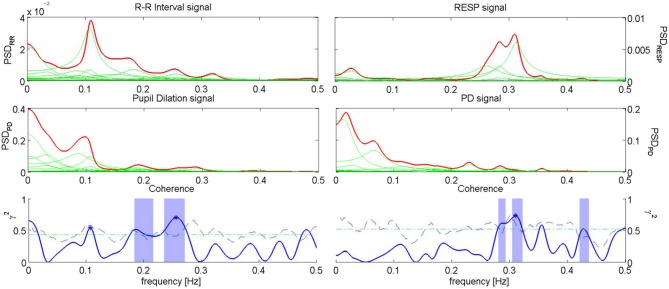
**Coherence analysis for PD and RR signal for the subject “sbj05,” during “Anger” event (left), and coherence analysis for PD and RESP for the subject “sbj15,” during “Happiness” event (right).** On the first row, the PSD of RR (left) and RESP (right) are depicted; on the second row, the PSDs of the PD for the two analysis are shown; on the third row, the plots show the Coherence between RR series and PD (left), and the Coherence between RESP signal and PD (right).

An example of Coherence analysis between PD, RR intervals and RESP is presented in Figure 5. The analysis between PD and the RR signal is shown on the left, while the analysis between PD and RESP is on the right.

In Table 5 the Coherence computed at the considered frequency bands is reported for each experimental condition, along with the number of subjects showing Coherence above threshold. For the Coherence between PD and the RR signal we considered two frequency bands, i.e., LF and HF; for the Coherence between PD and RESP, only the HF band was taken into account.

**Table 5:** **Mean values and standard errors of Coherence and number of subjects showing Coherence above statistical threshold**.

**γ^2^ (s.d.) *N*_θ_/*N***	**Baseline**	**Happiness**	**Anger**	**Sadness**
RR-PD, LF	0.502 ± 0.157	0.559 ± 0.225	0.547 ± 0.172	0.491 ± 0.213
	3/13	4/13	5/13	4/13
RR-PD, HF	0.647 ± 0.136	0.574 ± 0.133	0.625 ± 0.129	0.612 ± 0.124
	4/13	9/13	9/13	10/13
RESP-PD	0.688 ± 0.128	0.605 ± 0.146	0.617 ± 0.174	0.636 ± 0.192
	10/13	9/13	10/13	10/13
RR-RESP	0.888 ± 0.145	0.858 ± 0.113	0.774 ± 0.217	0.860 ± 0.083
	12/12	4/12	5/13	13/13

We indicated with γ^2^ the mean value of the Coherences; with s.d. the standard deviation of the Coherences; with N_θ_/N the rate of the subjects showing Coherence above statistical threshold.

As an overall results, it is possible to see that during “Baseline” the average Coherence shows higher values at HF and lower values at LF. The Coherence analysis between PD and the RR signal shows on average 4 Coherences above threshold in the LF band out of 13 subjects. In the HF band we reported 10 subjects out of 13 showing a Coherence above threshold during “Sadness,” and 9 subjects out of 13 during “Happiness” and “Anger”. For “Baseline” we found an above threshold Coherence in the HF band for 4 subjects out of 13, even though the average Coherence is higher than the other conditions: this result might be due to the method used to assess the significance zero level, which in some cases seems to be too conservative. In the analysis between PD and RESP, a higher Coherence has been reported (10 out of 13 above threshold for “Baseline,” “Anger,” and “Sadness,” and 9 out of 13 for “Happiness”).

**Figure 6:**
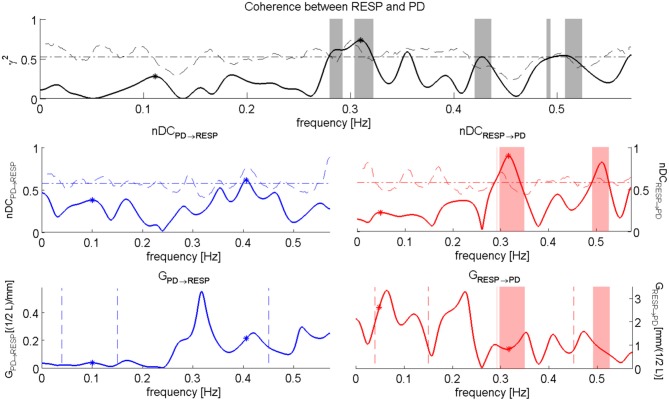
**nDC analysis between RESP and PD for the subject “sbj11,” during “Baseline” event.** The Coherence between RESP and PD is depicted on the top; in the middle we have the normalized direct Coherence considered from PD to RESP, i.e., nDC_PD→RESP_ (left), and the normalized direct Coherence considered from RESP to PD, i.e., nDC_RESP→PD_ (right); in the bottom the plots show the Gains for the respective transfer function, i.e., *G*_PD→RESP_ (right) and *G*_RESP→PD_ (left).

We computed also the nDC and the Gain of the related transfer functions. A graphical representation of the performed analysis is in Figure 6 while numerical results are presented in Table 6.

**Table 6:** **Mean values and standard errors of the nDC and the Gain of the transfer function, and the number of subjects showing nDC above statistical threshold**.

**nDC_*i* → *j*_ (s.d.) *G_i → j_ N_θ_/N***	**Baseline**	**Happiness**	**Anger**	**Sadness**
RR → PD, HF	0.797 ± 0.054	0.623 ± 0.065	0.722 ± 0.121	0.646 ± 0.103
	1.496 6/13	2.366 7/13	1.498 5/13	1.942 8/13
PD→RR, HF	0.685 ± 0.139	0.614 ± 0,180	0.706 ± 0.090	0.711 ± 0.146
	0.171 6/13	0.124 7/13	0.374 6/13	0.193 6/13
RESP→PD	0.756 ± 0.143	0.626 ± 0.167	0.662 ± 0.168	0.657 ± 0.145
	0.780 10/13	**1.398**^†^ 9/13	0.735 9/13	0.612 8/13
RESP→RR	0.927 ± 0.071	0.879 ± 0.101	0.841 ± 0.201	0.904 ± 0.082
	0.434 13/13	0.365 13/13	0.305 12/13	0.351 13/13

*Statistically significant differences were bold typed*.

A dagger (

† *) indicates a p-value < 0.016*.

*We indicated with nDC_i→ j_ the mean value of the normalized Directed Coherence; with s.d. the standard deviation of nDC; with G_i→ j_ the Gain of the transfer function from *i* to *j*; with N_θ_/N the rate of the subjects showing nDC above threshold*.

**Figure 7:**
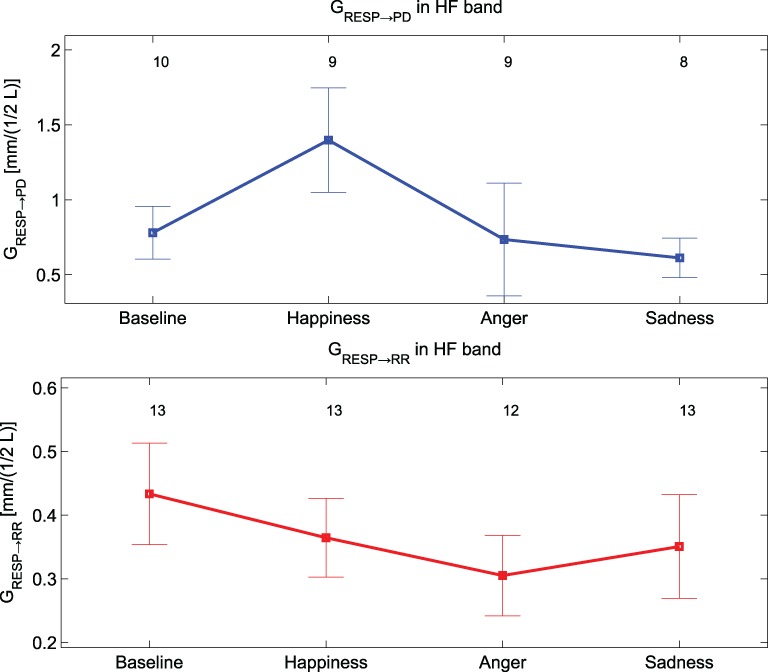
**Mean values and standard errors of *G*_Resp→ PD_ (at the top, in blue) and of *G*_RESP→ RR_ (at the bottom, in red)**.

Only *G*_RESP→PD_ shows significant differences [*F*_(3, 36)_ = 4.9, *p*-value < 0.05] within groups. The *post-hoc* analysis reveals significant differences between “Baseline” and “Happiness” (*p*-value < 0.01*6*). This index is the only bivariate index showing high significance for the experimental conditions of our protocol, and it's the only one which distinguishes emotional states with an opposite connotation. The results of the analysis between PD and the RR signal indicate weak coupling. At LF the rate of subjects showing nDC above threshold for both the transfer functions was low and in some conditions none of the subjects showed above threshold nDC (results not shown). At HF no clear directionality in the linear coupling can be stated: nDC is above threshold on average for the same number of subjects for both the transfer functions. As expected, in the analysis for PD and RESP, a higher number of subjects shows nDC_RESP→PD_ above threshold. Moreover, *G*_RESP→PD_ shows an interesting trend in Figure 7: once compared with *G*_RR→RESP_, *G*_RESP→PD_ shows higher values during “Happiness” with respect to the other conditions, in particular to “Anger” and “Sadness”.

**Figure 8:**
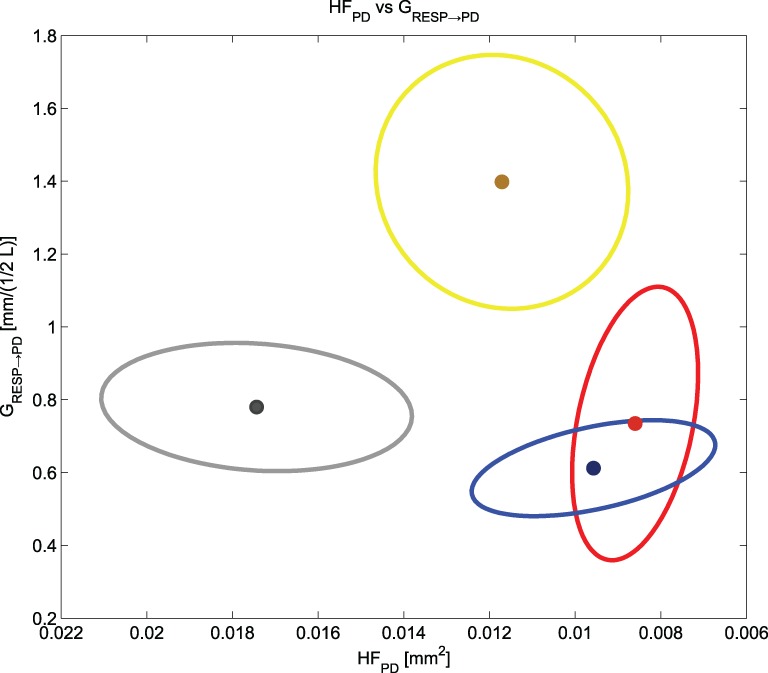
**Means and standard errors of HF_PD_ and *G*_RESP → PD_ for all the experimental conditions**. We have in gray “Baseline,” in yellow “Happiness,” in red “Anger” and in blue “Sadness”.

We noticed that HF_PD_ and *G*_RESP→PD_ cluster and separate the different experimental conditions (see Figure 8). While HF_PD_ shows a correlation with general activation, *G*_RESP→PD_ decreases at negative events and increases at the positive one: this finding is key for a prospective classification of different emotional events. A two-dimensional plot of the means and the standard errors of HF_PD_ and *G*_RESP→PD_ is shown in Figure 8, representing the experimental conditions considered in our protocol.

### 3.3. Discriminant analysis

In accordance to the results just presented, we chose the indices with highest statistical power. In Table 7 *S*e and *S*p of the discriminant analysis are reported. The chosen indices were HF power in normalized units and LF/HF ratio for RR signal, and HF absolute power, VHF_[2.5–5]_ and *G*_RESP→PD_ for PD. Also a linear combination of the PD indices, whose weights were obtained by a Principal Component Analysis (PCA), was included in the analysis: this feature obtained overall good performances, mostly in term of Sensitivity. This result suggests that including PD indices in emotionally characterized or stressful contexts might help to distinguish arousing events from resting conditions.

**Table 7:** **Sensitivity (*Se* = TPR) and Specificity (*Sp* = 1-FPR) of each feature when comparing “Baseline” (B) with respectively “Happiness” (H), “Anger” (A) and “Sadness” (S)**.

***Se, Sp***	**B-H**	**B-A**	**B-S**
HF_HRV_ norm	0.538, 0.923	0.615, 0.846	0.769, 0.692
LF/HF_HRV_	0.308, 0.846	0.385, 0.387	0.077, 1.000
HF_PD_	0.461, 0.692	0.615, 0.769	0.538, 0.846
VHF_[2.5–5]_	0.769, 0.461	0.615, 0.769	0.615, 0.615
*G*_RESP→PD_	0.461, 0.385	0.615, 0.615	0.692, 0.385
Lin. Comb._PD_	0.769, 0.538	0.769, 0.692	0.692, 0.615

## 4. Discussion

This work focuses on mathematical methods for PD signal processing aimed at estimating novel markers of autonomic activity and investigates PD dynamic changes during a psychophysiological study, in particular during emotionally characterized events compared with a general relaxation/deactivation condition. The analysis was performed through the following multiple steps: (1) we implemented an *ad hoc* reconstruction of the signal to recover as much information as possible during the blinking events, a well-known limitation in the analysis of PD; (2) we explored PD information content through classical statistical indices; (3) we performed a spectral analysis of the signal, comparing the results with respect some classic autonomic indices from RR signal and Respiratory signal; (4) we conducted a preliminary spectral analysis at higher frequencies, from 0.45 to 5 Hz; (5) we performed a Coherence analysis to explore the mutual influences of PD, RR signal and RESP during autonomic control.

The first step was to provide an algorithm for the reconstruction of the PD signal during blinking events and movement artifacts. The adopted procedure allowed to analyze PD at high frequencies, hence exploring the information carried by fast oscillations, and to improve the analysis of the signal at low frequencies, providing an estimation of the missing data as close as possible to the underlying dynamics of the observable data. From the reconstructed signals we computed time domain aggregate features. We observed an overall decrease in variability during triggering events. Although it was not sufficient to significantly distinguish emotional conditions from “Baseline,” this trend is in agreement with a common behavior of other physiological signals during psychophysiological events (Caldirola et al., 2004; Rainville et al., 2006).

As firstly hypothesized by Borgdorff (1975) and then supported in successive studies (Yoshida et al., 1994; Calcagnini et al., 2000), ANS modulation of PD dynamics is reflected in oscillations classically referred to the autonomic control of cardiorespiratory activity. Moreover, these oscillations could be linearly and mutually coupled to cardiorespiratory signals, which in turn are related to ANS dynamics. The autonomic control on the pupillary system might be driven by similar autonomic pathways that modulate heartbeat dynamics, but this does not exclude that the cardiorespiratory activity itself might be reflected on PD and might influence PD dynamics, either strengthening or inhibiting the more direct ANS influence on PD.

In this work we therefore explored the spectral components of PD in frequency ranges classically related to the ANS control of cardiorespiratory activity, i.e., LF and HF, computing widespread features. The observed trends of PD and RR series features confirm similar aggregate behavior for PD and RR signal. These patterns reflects similar autonomic modulation at HRV frequency bands for both pupillary and cardiovascular systems during emotional triggering events. The analysis of Coherence clarifies the nature of this behavior.

The results of Coherence analysis support the idea of a coupling between PD and cardiorespiratory activity, mostly for the respiratory influence on PD. In particular, results regarding the Coherence in the HF band are consistent with previous findings (Calcagnini et al., 2000) both in terms of Coherence values and rate of Coherence values above statistical threshold, and confirm the hypothesis that a rhythmic impulse coming from the respiratory centers drives fluctuations in a wide range of organs, and among them the pupillary system (Borgdorff, 1975). This hypothesis is further confirmed by high values of nDC_RESP→PD_ and a high number of subject showing nDC_RESP→PD_ above threshold. We were not able to establish a specific directionality in the coupling between PD and RR in the HF band. On the other hand, for RESP and PD the overall value of Coherence and nDC indicate that there is a relevant linear coupling, and that a similar autonomic modulation is driven by respiratory rhythms on both PD and HRV. These results, along with the results from the spectral analysis, support the hypothesis that there is a direct influence of RESP to PD. The nature of this connection might be different from the coupling between RESP and HRV directly affecting different autonomic pathways.

The low value of Coherence and nDC, as well as the unclear directionality between HRV and PD at LF could be explained by different hypotheses. Although a large part of the power content of PD is at low frequencies, these dynamics might not reflect autonomic control on the pupillary system, or at least an autonomic response to particular triggering events such as emotionally characterized events. Moreover, these dynamics show a weak linear coupling with HRV LF rhythms, as suggested by Calcagnini et al., (2000). A second hypothesis is that in these particular experimental conditions the sympathetic system is not stressed enough to evoke a clear and visible response. A further hypothesis to take into account is that the presence of other sources of fluctuations, such as *hippus* and accommodation (Charman and Heron, 2007), which could overlap in the LF band, may have created distortion phenomena difficult to quantify and eliminate.

As further outcome, we explored PD dynamics at higher frequencies (from 0.45 to 5 Hz). These oscillations have trends comparable to those in PD total variance and HF power. The highly significant differences between “Baseline” and “Anger” and the similar behavior of the signal at higher frequencies support the hypothesis of a direct central autonomic control on PD, which reflects also the response of the ANS to emotional triggering events. The HF_PD_ and VHF_PD_ components provides characterizing features, which might lead to propose PD-based markers in stressful, emotional or arousing events. For this reason, it would be interesting to explore more deeply the role of the central autonomic network as reflecting in PD responses during emotional events.

In addition, to deepen the classification prospect of PD features might improve nowaday classification performances and candidate PD as a new important signal in recognition and classification of emotional conditions in different research fields. In particular, the possibility to evaluate affective states from PD might lead to interesting developments for communication applications: as a contactless autonomic correlate, PD could cover a major role in the detection of arousing events elicited by audio-visual contents.

The results of our analysis show that the differences between the experimental conditions were reflected on PD indices and confirmed by the level of sensitivity and specificity of these indices in distinguishing a baseline state from the emotional events. For this reason, classification performances of these new indices need to be explored in future works. The influence of RESP to PD and the weak or absent linear coupling between HRV and PD in the LF band are other relevant outcomes of this work and confirm the findings previously reported in the literature. An important advance would be to evaluate the effect of known and controlled ANS changes on RR intervals, RESP and PD coupling. Importantly, *G*_RESP→PD_ seems to be related to the valence of the emotion, although further confirmations are required. In addition, VHF_[2.5–5]_ results might indicate that the ANS modulates PD at higher frequencies, and that these oscillations might be a reflection of central autonomic activity directly influencing PD. Exploring and studying in depth higher frequencies of PD, as well as its very LF oscillations such as *hippus*, is another future developments of this work. Some other major future directions include the study of non-linearity and non-stationarity of PD, possibly estimating time varying monovariate and multivariate indices through stochastic modeling of the signal (e.g., multivariate point-process models).

### Conflict of interest statement

The authors declare that the research was conducted in the absence of any commercial or financial relationships that could be construed as a potential conflict of interest.

## Footnotes

^1^The valence represents the degree of pleasure or displeasure an event or a stimulus is able to elicit. The arousal determines the degree of activation in response of an event or a stimulus (Lang, 1995).
